# Research on the effects of GLP-1 receptor agonists in treating cognitive dysfunction and gait disorders in elderly patients with diabetes

**DOI:** 10.3389/fphar.2025.1607443

**Published:** 2025-06-24

**Authors:** Yang Yang, Lifeng Chen, Yanjun Zhang, Wenqian Fu, Dandan Liu, Tianyu Jiang

**Affiliations:** ^1^ Department of Neurology, The 2nd Medical Center of Chinese PLA General Hospital, Beijing, China; ^2^ Department of Neurosurgery, The 1st Medical Center of Chinese PLA General Hospital, Beijing, China; ^3^ Department of Rehabilitation Medicine, The 2nd Medical Center of Chinese PLA General Hospital, Beijing, China

**Keywords:** GLP-1R agonist, diabites mellitus, cognitive functions, gait disorder, Parkinson's disease

## Abstract

Elderly patients with Type 2 Diabetes Mellitus (T2DM) often experience cognitive dysfunction and gait disorders, which significantly affect their quality of life and daily function. Glucagon-like peptide-1 receptor agonists (GLP-1RAs), known as “wonder drugs for weight loss,” have shown potential not only for glycemic control but also for improving cognitive function and gait disturbances. GLP-1RAs may exert their effects through various mechanisms, including promoting neurogenesis and synaptic plasticity in the brain, as well as reducing neuroinflammation, thereby improving cognitive and gait impairments either directly or indirectly. This review aims to explore the role of GLP-1RAs in the treatment of diabetes-related cognitive dysfunction and gait disturbances, as well as their potential mechanisms, providing a theoretical basis for clinical treatment.

## 1 Introduction

Epidemiological surveys indicate that diabetes mellitus (DM) is one of the most common chronic diseases, with China having the highest number of diabetes patients in the world. The prevalence of diabetes among adults has reached 11.9%, and it is as high as 28.8% among individuals over the age of 70 in China (National Health Commission of the People’s Republic of China and Bureau of Disease Prevention and Control, 2021). Type 2 Diabetes Mellitus (T2DM) is a metabolic disease, and its complications, cognitive dysfunction and gait disorders, are two critical clinical issues. Cognitive dysfunction is prevalent among elderly T2DM patients, manifesting as declines in memory, attention, executive function, visual function, and processing speed, and can even lead to dementia (Zhang et al., 2023). Gait disorders increase the risk of falls and limit patients’ mobility, both of which significantly impact patients’ quality of life.

In recent years, glucagon-like peptide-1 receptor agonists (GLP-1RAs), a new class of antihyperglycemic and weight-loss medications, have attracted widespread attention due to their remarkable blood sugar-lowering effects. GLP-1RAs mimic the action of endogenous glucagon-like peptide-1, enhancing insulin secretion and inhibiting glucagon secretion, thereby lowering blood glucose levels. Besides their role in blood glucose control, more and more evidences suggest that GLP-1RAs may have a positive impact on cognitive function and gait stability in patients with T2DM.

The literature reviewed research on the effects of GLP-1 receptor agonists in treating cognitive dysfunction and gait disorders in elderly patients with Diabetes. In this review, published articles from 2012 to 2024 were examined involved the randomized control trial (RCT), observational studies and meta studies. This review aims to explore the research progress on the therapeutic effects of GLP-1RAs in addressing cognitive and gait disorders in T2DM patients, as well as their potential mechanisms of action. It elaborates on the direct effects of GLP-1RAs on the central nervous system, including promoting neurogenesis, enhancing synaptic plasticity, reducing neuroinflammation, and alleviating oxidative stress, as well as the mechanisms through which GLP-1RAs may improve gait disorders by influencing motor control areas in the central nervous system. Furthermore, this review discusses the signaling pathways through which GLP-1RAs exert their effects, which play a key role in neuroprotection and cognitive function improvement. By comprehensively analyzing existing clinical trials and basic research, this review will provide a new perspective on the role of GLP-1RAs in treating cognitive and gait disorders related to T2DM, guiding future research directions and potential therapeutic strategies.

## 2 Overview of GLP-1 receptor agonists (GLP-1RAs)

GLP-1 (Glucagon-like Peptide-1) is an endogenous hormone secreted by the intestine. Upon binding to GLP-1 receptors on pancreatic β-cells, it activates adenylate cyclase, increasing intracellular cAMP levels, which in turn activates protein kinase A (PKA). The activation of PKA leads to the opening of L-type voltage-dependent calcium channels and the closing of KATP channels, resulting in cell membrane depolarization and calcium influx, ultimately promoting the exocytosis of insulin granules and completing the insulin secretion process, thereby playing a role in the treatment of type 2 diabetes (T2DM) (Erbil et al., 2019).

GLP-1RAs are a class of medications that mimic the action of GLP-1, lowering blood glucose levels through several mechanisms, including enhancing insulin secretion, inhibiting gastric emptying, increasing satiety, suppressing glucagon secretion, increasing glucose uptake and utilization by peripheral tissues, and protecting pancreatic β-cells. Currently, GLP-1RAs approved for clinical use in China include Liraglutide, Dulaglutide, Semaglutide, Lixisenatide, Exenatide, Bydureon, and Pegylated Liraglutide. Based on their molecular structure, GLP-1RAs can be classified into those based on human GLP-1 structure and those based on Exendin-4 structure. The former includes Liraglutide, Dulaglutide, and Bydureon, which share a high homology to human GLP-1 (≥90%), with Bydureon having 100% homology to human GLP-1. The latter includes Exenatide, Lixisenatide, and Pegylated Liraglutide, which have approximately 50% homology to human GLP-1. Furthermore, GLP-1RAs can be categorized according to pharmacokinetic properties into short-acting, long-acting, and ultra-long-acting formulations. Short-acting formulations, such as Bydureon, Exenatide, and Lixisenatide, typically require subcutaneous injections 1–3 times daily. Long-acting formulations, like Liraglutide, generally require one injection per day, while ultra-long-acting formulations, including Dulaglutide, extended-release Exenatide, and Pegylated Liraglutide, are usually administered once a week (Chinese Society of Endocrinology and Chinese Society of Diabetes, 2020).

GLP-1RAs not only act on the pancreas but also exert effects in the heart, coronary arteries, kidneys, gastrointestinal tract, as well as brain regions such as the hypothalamus and hippocampus, indicating their wide-ranging effects (Yaribeygi et al., 2021). Recent studies have found that GLP-1RAs may cross the blood-brain barrier and directly affect the central nervous system, improving cognitive function (Li et al., 2021). Research has shown that, compared to other oral hypoglycemic agents, Liraglutide significantly increases activation in brain regions and enhances cognitive function in T2DM patients (Mahapatra et al., 2022). Some studies suggest that Semaglutide can improve cognitive function in patients with neurodegenerative diseases and may promote neuroprotection. Additionally, the improvement of gait disturbances associated with GLP-1RAs may relate to their influence on motor control and coordination, as well as potential protective effects within the central nervous system. Research has shown that Lixisenatide exhibits neuroprotective properties in a mouse model of Parkinson’s disease, and early Parkinson’s patients receiving Lixisenatide treatment for 12 months experienced a significant slowing of motor symptoms progression compared to those on placebo (Meissner et al., 2024) Table 1.

**TABLE 1:** The effects of various GLP-1R agonists up on brain mechanisms.

Drug	Effect of attenuating neuroinflammation	Effect of improving cognitive function	Effect of improving gait stability	Reference
Liraglutide	+	+		Mahapatra et al. (2022), Zeng et al. (2020)
Dulaglutide	+	+		Guan et al. (2023)
Bydureon	+	+	+	Holscher (2024)
Exenatide	+	+	+	Li et al. (2012)
Lixisenatide	+		+	Meissner et al. (2024)
Semaglutide	+	+		Nowell et al. (2023), Holscher (2024)
Dual GLP-1/GIP receptor agonist tirzepatide	+	+	+	Chuang et al. (2024)

## 3 Mechanisms of action of GLP-1 receptor agonists in the central nervous system

GLP-1 receptors are widely distributed in the central nervous system, particularly in key brain regions such as the hippocampus, thalamus, striatum, amygdala, hypothalamus, and temporal cortex, which are critical for the regulation of cognitive functions (Wang et al., 2021). Notably, the expression of GLP-1 receptors in the hippocampus has a significant impact on memory and learning processes. Therefore, GLP-1 receptor agonists (GLP-1RAs) may potentially improve cognitive function in patients with diabetes by activating these receptors, and this effect is not limited to blood glucose control, but also includes direct neuroprotection and cognitive enhancement effects on the central nervous system. GLP-1RAs may exert neuroprotective effects that improve cognition through mechanisms such as promoting neurogenesis, inhibiting neuroinflammation, reducing oxidative stress, and enhancing synaptic plasticity (Guan et al., 2022).

### 3.1 Neuroprotective effects

#### 3.1.1 Promotion of neurogenesis or reduction of neuronal injury

Research suggests that improvements in memory function are associated with stable neural connectivity, neurite outgrowth, and the maturation of dendritic spines. Exendin-4 has been shown to contribute to the formation of stable neural structures and promote neurite outgrowth and dendritic spine maturation of hippocampal and cortical neurons under conditions of metabolic imbalance (Cembrowski et al., 2016). Studies on GLP-1RAs in neurogenesis are still in relatively early stages. Dulaglutide treatment has been reported to significantly reduce neuronal injury in the hippocampus of rats with vascular dementia (VD) and decrease the proliferation of microglia and astrocytes. Dulaglutide may alleviate learning and memory deficits as well as neuronal injury in VD rats by reducing apoptosis, modulating autophagy, and activating the PI3K/Akt/mTOR signaling pathway in neurons (Guan et al., 2023). Moreover, in rat models and following oxygen-glucose deprivation (OGD) damage, Liraglutide treatment has been shown to inhibit apoptosis and promote neuronal survival (Zeng et al., 2020).

#### 3.1.2 Inhibition of neuroinflammation

GLP-1RAs exert anti-inflammatory effects in the central nervous system by activating GLP-1 receptors in the brain, leading to the indirect inhibition of pro-inflammatory cytokine production through various neuronal signaling pathways, including adrenergic and opioid receptors. Research from Daniel Drucker’s team has found that the anti-inflammatory effects of GLP-1RAs depend on GLP-1 receptors in the central nervous system. GLP-1RAs can cross the blood-brain barrier and activate GLP-1Rs in the brain, which subsequently inhibits the release of pro-inflammatory cytokines, particularly reducing the production of tumor necrosis factor-alpha (TNF-α) triggered by Toll-like receptor (TLR) activation. Neuroinflammation has emerged as a significant risk factor contributing to cognitive impairment in various neurodegenerative diseases, and its role in type 2 diabetes mellitus (T2DM) has gained increasing attention (Wong et al., 2024). Studies have confirmed that metabolic dysregulation in the brain of mice with diabetic encephalopathy (DE) can induce neuroinflammation, with the release of numerous inflammatory factors damaging cerebral vasculature and hippocampal neurons, ultimately leading to cognitive dysfunction. Pharmacological agents can mitigate the inflammatory response in DE rat brains, thereby improving learning and memory abilities (Zhang et al., 2024). Another study has shown that Liraglutide treatment significantly alleviates brain edema, reduces inflammatory responses, promotes tissue recovery, and enhances prognosis after brain injury, revealing that Liraglutide exerts neuroprotective effects following neonatal hypoxic-ischemic brain injury via the PI3K/Akt/glycogen synthase kinase-3β (GSK3β) pathway (Zeng et al., 2020).

#### 3.1.3 Reduction of oxidative stress

Diabetes and hyperglycemia promote oxidative stress responses in multiple organs, leading to tissue damage. Numerous studies emphasize that GLP-1 receptor agonists (GLP-1RAs) have shown promising therapeutic results in alleviating oxidative damage. Neuronal apoptosis induced by oxidative stress is associated with the development of cognitive impairment. Oxidative stress can alter the expression of Bcl2 (B-cell lymphoma 2) family proteins, which are involved in anti-apoptotic processes and can regulate mitochondrial permeability by altering transition pores (Xue et al., 2019). GLP-1RAs help increase the production of antioxidant molecules, thereby combating cell damage caused by oxidative stress. GLP-1 receptors are widely expressed in the brain and spinal cord, and studies have shown that the long-acting GLP-1 receptor agonist exendin-4 (Ex-4) has neuroprotective effects in various animal models of neurodegenerative diseases, including stroke, Parkinson’s disease, and Alzheimer’s disease. Ex-4 has been demonstrated to possess neurotrophic effects in NSC-19 cells, enhancing choline acetyltransferase (ChAT) activity and protecting cells from hydrogen peroxide-induced oxidative stress and astrogliosis-induced apoptosis (Li et al., 2012).

#### 3.1.4 Enhancement of synaptic plasticity

GLP-1RAs can promote the formation of synapses in the cortical and hippocampal regions, enhancing synaptic plasticity and thereby improving cognitive function (McClean and Hölscher, 2014). GLP-1 (9–36) is a natural cleavage product of GLP-1, and studies have shown that GLP-1 (9–36) enhances hippocampal synaptic plasticity, which may be attributed to its GLP-1RA effects, as well as its regulation of dendritic membrane excitability and protein synthesis (Day et al., 2017). Research indicates that activation of GLP-1 receptors has a protective effect against cytokine-mediated apoptosis and may stimulate neurogenesis (Erbil et al., 2019). GLP-1RAs may promote the growth and maintenance of neuronal synapses by increasing levels of neurotrophic factors such as brain-derived neurotrophic factor (BDNF) (Gonzales et al., 2020).

### 3.2 Mechanisms of GLP-1RA on gait disorders

#### 3.2.1 Neural basis of gait control

The central nervous system plays a significant role in gait coordination (Takakusaki, 2017). Gait control is an important aspect of nervous system function, involving the coordinated work of multiple brain regions, including the cerebral cortex, basal ganglia, cerebellum, brainstem, and spinal cord. These regions interact through complex neural networks to ensure stability, coordination, and adaptability during walking (Montero-Odasso et al., 2017). Studies have found that cognitive and motor regulation are controlled by the same neural networks in the brain, primarily located in the frontal and temporal lobes. The frontal lobe is a critical area responsible for executive functions, such as planning, decision-making, and social behavior, while the temporal lobe is associated with memory, language, and auditory processing (Chen et al., 2018).

#### 3.2.2 Direct effects of GLP-1RA on gait disorders

GLP-1 receptor agonists (GLP-1RA) may positively impact gait disorders by improving insulin resistance and inflammation. Given that GLP-1 receptors are widely distributed in the brain, GLP-1RAs can act directly on the central nervous system, enhancing the function of areas related to gait and motor control. Studies in experimental animal models have shown that GLP-1 analogs not only delay cognitive decline but also significantly improve motor deficits in Parkinson’s disease (PD) mice (Badawi et al., 2019). In research on Parkinson’s disease rat models, GLP-1 interventions have been found to significantly improve motor dysfunction and reduce damage to dopaminergic neurons (Elbassuoni and Ahmed, 2019). Other studies indicate that diabetes may exacerbate PD symptoms, whereas GLP-1 can improve PD symptoms in a dose-dependent manner by increasing dopamine levels in the striatum through its direct antioxidant and anti-inflammatory effects, as well as indirectly by lowering blood glucose levels (Filchenkoi et al., 2018). It is noteworthy that the neuroprotective effects of GLP-1 do not rely on the levels of blood glucose control (Efthymiou et al., 2023).

#### 3.2.3 Indirect effects of GLP-1RA on gait disorders

GLP-1RAs may improve gait control by enhancing central nervous system function and indirectly affecting gait through improvements in peripheral nerve function and vascular health. In diabetic patients, sensory impairments (such as loss of vibration and protective sensations and/or neuropathic ulcers), reduced lower limb strength, and central nervous system damage can all lead to the development of gait disorders. Type 2 diabetes mellitus (T2DM) patients might experience gait abnormalities due to diabetes-related peripheral neuropathy (DPN). DPN typically begins with sensory neuropathy and may progress to motor neuropathy, leading to muscle atrophy and reduced joint mobility, thereby affecting gait. Studies have shown that even in T2DM patients without obvious symptoms and signs of diabetic peripheral neuropathy, early-stage DPN may be associated with declines in gait function. Although gait abnormalities can occur throughout the course of diabetes and worsen with disease severity, damage to large fiber nerves is often asymptomatic in early stages, with gait abnormalities increasing as diabetes severity escalates (Huang et al., 2022).

### 3.3 Regulation of GLP-1RA signaling pathways

GLP-1RAs involve multiple signaling pathways in their neuroprotective effects within the central nervous system. They activate several kinases signaling pathways associated with GLP-1 receptors (GLP-1R), such as cyclic adenosine monophosphate-protein kinase A (cAMP-PKA) (Guan et al., 2023), phosphoinositide 3-kinase/protein kinase B (PI3K/AKT) (Qi and Lixin, 2022), extracellular signal-regulated kinase (ERK), and mitogen-activated protein kinase (MAPK) (Colin et al., 2023), to regulate neurotransmitter transmission and exert neuroprotective effects. Activation of these signaling pathways helps reduce inflammation, diminish oxidative stress, inhibit apoptosis, decrease DNA damage, and promote neuronal repair, ultimately achieving neuroprotection. Research indicates that when GLP-1 binds to its receptor, it activates intracellular adenylate cyclase, leading to an increase in intracellular cyclic adenosine monophosphate (cAMP) levels. cAMP acts as an important second messenger within cells, and its increase subsequently activates protein kinase A (PKA) and phosphoinositide 3-kinase (PI3K) pathways. Since the pathways activated by GLP-1 overlap with those activated by insulin, including the MAPK pathway, which involves various cellular responses such as growth, differentiation, and survival, GLP-1RA can help restore cellular sensitivity to insulin that may have been compromised due to insulin resistance. In other words, GLP-1RAs can reactivate intracellular signaling pathways that have become dysfunctional due to insulin resistance. While GLP-1RAs and insulin act through different receptors, they share downstream signaling pathways, and by activating these overlapping pathways, GLP-1RAs can aid in restoring insulin signaling that is potentially impaired in patients with type 2 diabetes (Chen et al., 2024).

Additionally, GLP-1 receptor agonists (GLP-1RAs) may regulate appetite and body weight by directly acting on neurons in the hypothalamic regions, such as GLP-1R-positive neurons in the lateral septum (LS), indicating that GLP-1RAs may exert effects through multiple mechanisms within the central nervous system (Chuang et al., 2024).

## 4 Summary and outlook

GLP-1 receptor agonists (GLP-1RAs) are a novel class of hypoglycemic agents that improve cognitive function in individuals with type 2 diabetes through various mechanisms, including promoting neurogenesis, inhibiting neuroinflammation, reducing oxidative stress, and enhancing synaptic plasticity. Similarly, GLP-1RAs enhance central nervous system function to improve gait, while indirectly improving gait disturbances by enhancing peripheral nerve function and vascular health. Although the mechanisms of GLP-1RAs are complex and not yet fully understood, existing research suggests significant potential for their use in treating diabetes and its related neurological complications. Future studies will further elucidate their specific roles in improving cognitive function and gait disturbances, as well as exploring their combination with other therapeutic methods to achieve optimal therapeutic outcomes.
